# Assessing Mothers’ Parenting Stress: Differences Between One- and Two-Child Families in China

**DOI:** 10.3389/fpsyg.2020.609715

**Published:** 2021-01-15

**Authors:** Guoying Qian, Jin Mei, Li Tian, Gang Dou

**Affiliations:** ^1^College of Preschool Education, Capital Normal University, Beijing, China; ^2^School of Psychology, Capital Normal University, Beijing, China; ^3^School of Education, Hubei University of Arts and Science, Xiangyang, China

**Keywords:** preschool children, two-child families, one-child families, parenting stress, mothers’ parenting stress

## Abstract

This study aimed to investigate mothers’ parenting stress and explore its relationship with associated demographic variables in two-child families involving preschool children. A sample of 621 two-child families and a comparison group of 319 one-child families from China participated in the study; the children were aged between 3 and 7. The results showed that (1) mothers of two-child families had higher parenting stress than those of one-child families; (2) within the two-child families, demographic variables, such as birth order, gender combination, and the age gap were found to have significant effects on maternal stress levels; and (3) in two-child families, families with an income of less than 3000 yuan had significantly higher maternal stress than families with an income of more than 6000 yuan.

## Introduction

Nearly all parents experience pressure as they attempt to meet the challenges of bringing up their children, especially in the preschool stage. This pressure may rise to comparatively high levels, owing to children’s moodiness and unreasonable demands, among other reasons (Koeske and Koeske, 1990). With the formal implementation of a universal two-child policy, two-child families have drawn increasing scholarly attention. The transition from one-child to two-child families has led to increasing economic burdens and restructuring of relationships within families. Additionally, obvious differences exist in raising two children as every child is unique. Therefore, parents have to face new challenges and more complex situations. In other words, compared with raising one child, parents with two children experience more pressure (Krieg, 2007).

Parenting stress, directly related to raising children, generally refers to a sense of strain that often prevents parents from leading a peaceful life. Such pressure derives from a feeling of decline and absence in parenting capacity, being constrained to play other life roles and lacking social support, as well as negative emotional experiences and states, such as anxiety, fear, worry, and exhaustion in parental practice (Abidin, 1995). Working women in China experience more pressure than men in the family because, as mothers, they also undertake the main responsibilities of child-rearing.

Abidin put forward a parenting stress model, pointing out that parenting stress leads to negative parenting behaviors, such as strict discipline and punishment, which exacerbates behavioral problems in children (Abidin, 1990; Neece et al., 2012; Crum and Moreland, 2017; Lohaus et al., 2018). Abidin (1990) states that parenting stress is caused by both parental characteristics and situational factors. Bronfenbrenner’s bioecological theory posits that people interact with the surrounding physical and sociocultural environment. It emphasizes that individual behavior is the result of the interaction between individuals and the environment (Bronfenbrenner, 1977). The law of family interaction proposed by the sociologist, Bossard (1945), postulates that family relationships in two-child families are more complicated and diverse than those of one-child families. In contrast to raising one child, a mother of two children needs to adapt to both children and establish essentially the same, but individual, parent–child relationships with each child. She also needs to correctly and smoothly deal with the relationship between the two siblings (Feng, 2015). Therefore, the number of children (Fulkerson et al., 2019), the traits of parents themselves, family situations, and various negative events in the living and working environment (Belsky, 1984; Evans and Kim, 2013; Loader et al., 2019) are risk factors for mothers’ parenting stress.

Researchers in China and abroad have found that multi-child mothers have higher parenting pressures than their one-child counterparts (Anthony et al., 2005; Hong and Liu, 2020). Mothers’ parenting stress and the nature of pressure also depend on the children’s characteristics and gender. In China’s long history of feudal civilization, the feudal ethical code centered on male projects’ profound influence on the old and even the modern society of China (Lu and Su, 2004; Sun, 2008). After birth, children are expected to have different roles depending on their gender, for example, boys are required to be more independent, resolute and brave, full of responsibility, and achieve fame, while girls should be gentle, virtuous, and industrious and thrifty in managing their households (Zhang, 2003). Therefore, mothers raising boys experience more stress (Fang et al., 2012; Liu and Wang, 2018). However, other researchers have found that parental stress is not related to the child’s gender (Li et al., 2005; Geng et al., 2009).

Children themselves are in a constant process of change as they grow older. Some researchers argue that mothers’ parenting stress decreases as children age and that mothers are more likely to experience greater adjustment stress when their children are in early childhood (Li et al., 2005). Mothers’ level of parenting stress remains relatively stable with children aged 3–6 (Hong et al., 2014). In contrast, studies of exceptional children have shown that mothers’ parenting stress increases with children’s age (Parkes et al., 2011), indicating that sample differences may have produced inconsistent results on the impact of children’s age on parenting stress.

Additionally, differences in sibling gender combination and the age gap between siblings also lead to different outcomes pertaining to parental stress, compared with one-child families. At present, most researchers have studied parental stress in two-child families from the perspective of sibling relations, but there are few studies on the influence of sibling gender combinations and sibling age gap on parenting stress. Given that parents’ psychological feelings in raising children differ on the basis of the child’s gender, there is an urgent need to further explore the impact of sibling gender combinations and age gap on parenting stress in two-child families.

The economic status of the parents is also related to the one-child mother’s parenting stress. For example, studies show that low-income mothers tend to have higher levels of parenting stress than parents who have more time and energy to spend with their children as a result of better economic status (Raikes and Thompson, 2005). As for educational level, studies have found that highly educated parents are more likely to adopt effective strategies to alleviate insecurities and anxieties generated during parenting and have more parenting knowledge and social resources. Thus, the higher the education level of the mother, the less parenting stress she experiences (Li et al., 2005). For two-child families, with the increase in household economic expenditure and the emergence of new family relations, the relationship between family income and parenting stress remains unclear, as does the relationship between parents’ educational background and parenting stress, both of which are worthy of further research.

In terms of the unique family planning policy in China, maternal parenting stress in two-child families has not received sufficient attention in previous studies. In addition, previous studies on mother’s parenting stress were mostly from the perspective of the child’s gender, age, and economic income associated with one-child families. Therefore, this study examines differences in mothers’ parenting stress in both one-child and two-child families and further explores the effects of sibling quantity, structural features (including birth order, combination of sibling gender, and age gap), mother’s educational background, and family income on mothers’ parenting stress.

## Materials and Methods

### Participants

In this study, cluster sampling was used to investigate families with children enrolled in two kindergartens in Nanchang city, of Jiangxi province. A total of 1031 questionnaires were distributed, but only 940 responses were usable after excluding invalid questionnaires (e.g., families with three children): 319 one-child families and 621 two-child families, including 302 first-born children (their ages ranging from 3 years, 2 months to 6 years, 6 months; birth intervals between these first-born children and their younger siblings ranged from 12 to 72 months) and 319 second-born children (their ages ranging from 3 years, 1 month to 6 years, 8 months; birth intervals between these second-born children and their older siblings ranged from 12 to 96 months). The mean age of the participants’ children was 4.8 (*SD* = 0.9), of which, 349 were from the kindergarten level 1 class, 267 from level 2, and 324 from level 3. Regarding gender, participants included 509 boys and 431 girls. Six percent of the mothers reported a combined income of less than 3000 yuan (*n* = 63), and 63% of mothers reported a combined income of more than 6000 yuan (*n* = 631). Twenty-six percent of the mothers had completed at least a university degree (*n* = 262), 28.8% of mothers had completed college (*n* = 262), 30% of mothers had a high school diploma (*n* = 300), and 15% of mothers had less than high-school-level education (*n* = 152). The kindergarten, teachers, and parents in the study provided signed informed consent in a written form in accordance with the Declaration of Helsinki before the study started.

### Measures

#### Parenting Stress

A revised Chinese version of the Parenting Stress Index-Short Form (PSI-SF) developed by Abidin (1990) and Yeh et al. (2001) was used in this study. The form included 12 items (e.g., “After having children, I can hardly do what I like.”) completed by the mothers. Participants responded on a five-point scale, ranging from 1 (strongly disagree) to 5 (strongly agree). Higher scores indicated that the mothers had higher parenting stress. Cronbach’s α was 0.89.

### Procedure

Questionnaires were sent to participants via WeChat and were completed online. The Ethics Committee for Scientific Research at the first author’s university approved the study.

### Data Analysis

Data were analyzed using SPSS 22.0. Pearson correlation analysis and multiple regressions were used to examine relationships between mothers’ parenting stress and their demographic variables.

## Results

### Number of Children and Structural Features

Two variables were controlled for: the mother’s educational level and the family’s economic income. The number of children was the independent variable and the mother’s parenting pressure was the dependent variable. On performing a hierarchical regression, the results indicated that the number of children had a significant predictive effect on the mother’s parenting pressure and the stress score of two-child mothers was significantly higher than that of only child mothers (as shown in Table 1).

**TABLE 1:** Comparison of the mothers’ parenting stress in only-child families and two-child families.

	*M* ± *SD*		β	*t*
Number of children	Only-child	29.50 ± 9.53	0.13	4.02***
	Two-child	32.55 ± 9.72		

***p < 0.001.

Table 2 shows the influence of the children’s birth order on the mother’s parenting stress in two-child families. By using an independent sample *t* test, we found that differences in birth orders were associated with differences in the mothers’ parenting stress. Mothers of first-born children scored significantly higher on parenting stress than mothers of second-born children [*t*(938) = 2.45, *p* < 0.05].

**TABLE 2:** Comparison of the mothers’ parenting stress in two-child families with children of different birth orders.

	*M* ± *SD*		*t*
Birth order	First-born	33.87 ± 9.85	2.45*
	Second-born	31.66 ± 9.30	

*p < 0.05.

A one-way ANOVA was conducted to examine the influence of sibling gender combination on mothers’ parenting stress in two-child families (Table 3). It was found that the main effect of sibling gender combination on mothers’ parenting stress was significant [*F*(3,617) = 3.568, *p* < 0.05, η*_*p*_*^2^ = 0.09]. Further *post hoc* tests showed that the mothers’ parenting stress score in a family with one son and one daughter was significantly lower than that of a family with children of the same gender, and there was no significant difference in mothers’ parenting stress between families with either two sons or two daughters.

**TABLE 3:** Comparison of sibling gender combinations of mothers’ parenting stress in two-child families.

		*M* ± *SD*	*F*	*Post hoc* comparison
Sibling gender combination	Male–Male	33.96 ± 9.74	3.568*	Female–Male < Male–Male*
	Female–Female	34.32 ± 8.09		Male–Female < Female–Female*
	Male–Female	31.69 ± 9.89		

*p < 0.05.

We conducted a univariate regression analysis to examine the predictive effect of the sibling age gap (the age of the first child minus that of the second child) on mothers’ parenting stress. As shown in Table 4, overall, the coefficient of determination equaled 0.02 (*R*^2^ = 0.02), indicating that the sibling age gap could effectively explain 1.6% of the variance in the mother’s parenting stress. The standardized regression coefficient, β, reached a significant level (β = −0.13, *p* < 0.01), indicating that the greater the age gap between siblings, the less the mother’s parenting stress. For first-born children, the results showed that the sibling age gap was not a significant predictor of maternal parenting stress. For second-born children, prediction of sibling age gap on maternal parenting stress was marginally significant (β = −0.12, *p* < 0.1).

**TABLE 4:** Univariate regression analysis of mothers’ parenting stress by sibling age gap.

Predictive variables	*N*	*B*	*R*^2^
Age gap of overall sample	621	−0.13**	0.02
First-born	302	–0.06	–0.00
Second-born	319	–0.12	0.01

**p < 0.01.

### Family Income and Mother’s Education Level

Table 5 shows a one-way ANOVA examining the main effects of two-child family economic income and mothers’ educational level on maternal parenting stress. We found that the main effect of family economic income on mothers’ parenting stress was significant, *F*(3,617) = 2.674, *p* < 0.05, η*_*p*_*^2^ = 0.08. Further *post hoc* comparisons showed that mothers’ parenting stress in families with an income of less than 3000 yuan was significantly higher than that of mothers in families with an income of more than 6000 yuan, and there were no significant differences in mothers’ parenting stress associated with other family income levels. We also found that the main effect of mothers’ education level on parenting stress was not significant, *F*(3,617) = 0.795, *p* > 0.05, η*_*p*_*^2^ = 0.00, and further *post hoc* tests showed that there were no significant differences in parenting stress among mothers with different educational backgrounds.

**TABLE 5:** Influences of family economic income and education levels on mothers’ parenting stress.

		*M* ± *SD*	*F*	*Post hoc* comparison
Family economic income	Less than 3000 yuan	35.81 ± 8.62	2.674*	Less than 3000 yuan > 6000–10,000 yuan* Less than 3000 yuan > More than 10,000 yuan**
	3000–5999	33.13 ± 10.29		
	6000–10,000 yuan	32.26 ± 8.66		
	More than 10,000 yuan	31.55 ± 9.72		
Education level of mother	Less than high school	33.15 ± 9.42	0.795	
	High school or GED	32.23 ± 9.87		
	College	33.18 ±10.13		
	University degree or higher	31.72 ± 9.17		

**p < 0.01, *p < 0.05.

## Discussion

The present research was framed within China’s universal two-child policy, providing unique empirical evidence regarding the difference between mothers’ parenting stress in one-child and two-child families, and the relationship between mothers’ parenting stress and the associated demographic variables. Major results showed that it is more stressful for mothers to raise two children than one, which is consistent with previous studies (Krieg, 2007). The greater stress may be due to: (1) Bossard’s law of family interaction that posits that family relations of two-child families will be more complicated and diversified than those of one-child families (Bossard, 1945). Two-child mothers need to learn to adapt to having two children and to establish a parent–child relationship that is essentially the same, but practically distinct, considering the individual personalities of the children. In addition, as a two-child mother, it is necessary to correctly handle both the parent–child relationship and the sibling relationship, as well as learn how to correctly treat and educate the two children in terms of norms, knowledge, and methods (Feng, 2015). (2) Mothers with two children require more time and energy to meet the demands of parenting. Many mothers feel discouraged and depressed because they have to sacrifice their time and freedom to be a parent. This intensifies their parenting stress. In addition, the cost of rearing is an important factor that affects fertility intention and behavior (Becker, 1960). The financial burden associated with child-rearing may inspire mothers to feel guilty about spending money or having fun, further increasing parenting stress (Chen, 2016).

As for sibling structure, this study found that in two-child families, sibling birth order had a significant impact on maternal parenting stress. Specifically, mothers experienced greater stress when parenting first-born children than second-born children. Thus, the mother’s parenting stress is greater when both children are of preschool age. Li et al. (2005) found that mothers’ parenting stress was associated with a child’s age. For example, parenting stress for mothers of 2-year-olds was significantly higher than that for mothers of 5-year-olds. Two-year-old children, in a stage of rapid development of self-awareness and cognitive ability, struggle with separation anxiety from their mothers and experience difficulty in adapting to kindergarten life. All the above situations intensify the mother’s parental stress (Li et al., 2005). As children reach the age of 5 and over, their abilities and psychological maturity gradually increase, and the parents’ parenting stress subsequently reduces. From what has been discussed above, the level of pressure for two-child mothers is high when a child is an infant or a toddler, and the other child is self-aware but unable to be completely independent.

Furthermore, the gender combination of siblings also has an important influence on the parenting stress of mothers. It was revealed that the parenting stress of mothers with both a son and a daughter was significantly lower than that of mothers with same-sex siblings. First of all, from the perspective of fertility options, having two children with different genders is the ideal choice for most Chinese parents at present (Wang, 2015; Hong and Zhu, 2017), as mothers with two children of different genders are likely to have a more positive parenting experience (Xu, 2017).

At the same time, because of the strong preference for male children in China, some families prefer sons over daughters. Some rural families even hold the view that if they do not have a son, they are failing to fulfill their personal reproductive mission (Xiao and Feng, 2010). Since the participants in this survey were from country areas, mothers with two daughters were more likely to experience higher levels of parenting stress. In addition, boys tend to have more conflicts with their mothers regarding discipline, which adds to the difficulty in parenting. Thus, boys consume more of their mothers’ energy, causing them greater stress. In China, raising a boy requires greater consideration of future costs, leading to a mother with two sons experiencing greater anxiety about the future (Qu et al., 2018). When it comes to siblings, studies have indicated that same-sex siblings are more likely to make social comparisons. Boys are more likely to compare themselves to their brothers when they are treated differently by their parents, which makes them feel more anxious and depressed (Buist et al., 2013). Similarly, girls are also concerned about the degree of intimacy and emotional involvement of the parents with their siblings, including the time spent together, communication, and the degree of attention. Therefore, girls are more likely to compete for parents’ affection (Deutz et al., 2014).

Furthermore, the sibling age gap has a significant impact on mothers’ parenting stress. Specifically, the larger the age gap, the less the parenting stress of mothers. Older siblings often play an exemplary role through social learning mechanisms, providing advice, help, and other resources to younger siblings (Zhao and Yu, 2017). Some even play the role of caregivers for younger siblings. Therefore, it is possible that as the age gap increases, the positive interaction between siblings can help release the mother’s time and energy as well as relieve the mother’s parenting stress. Moreover, raising two children requires huge reproductive costs, including economic and time costs (Hong and Zhu, 2017). If there is a small age gap between the two children, the fact that two children grow up at the same period will cause greater financial pressure on the mother, especially when both children are at an early school age. This situation means that mothers need to spend more time and energy taking care of the children, resulting in a higher level of parenting stress.

Furthermore, this study found that when the family income was 6000–10,000 yuan, the maternal stress in two-child families is higher than that of single-child families [*t*(374) = 4.93, *p* < 0.001]. It also demonstrates that in two-child families, there are significant differences in parenting stress among mothers with different family incomes, with the parenting stress of mothers in low-income families being significantly higher than that of mothers with decent family economic incomes, as is consistent with previous studies (Raikes and Thompson, 2005; Geng et al., 2009). On the one hand, this may be due to the fact that mothers with lower family incomes tend to face greater life pressure, which brings about a sense of urgency in life, affecting their own parenting stress. On the other hand, a mother engaged in work because of life pressure, in some cases, is likely to be too busy to spend time and energy on her child’s upbringing, increasing her own parenting stress.

This study confirms that in China, the parenting pressure for two-child mothers is higher than that of their one-child counterparts. Increasing family economic income and appropriately widening the age gap between siblings can effectively reduce mothers’ parenting stress and therefore be beneficial to a mother’s physical and mental health. However, the research subjects of this study were mainly mothers of preschool children, and few mothers of children of other ages were involved. Further research is needed to expand the scope of research subjects for verification of the findings. In addition, there are other factors that may influence parenting stress among two-child families (e.g., hukou, parents’ own sibling status, grandparents’ support), and the parenting pressure on the father also needs to be studied in the future.

## Data Availability Statement

The raw data supporting the conclusions of this article will be made available by the authors, without undue reservation.

## Ethics Statement

The studies involving human participants were reviewed and approved by the Scientific Research Ethics Committee of College of Preschool Education, Capital Normal University, China. The patients/participants provided their written informed consent to participate in this study. Written informed consent was obtained from the individual(s) for the publication of any potentially identifiable images or data included in this article.

## Author Contributions

GYQ designed the project and supervised the data collection. JM, GYQ, and GD collected and analyzed the data. GYQ and GD wrote the manuscript with input from all other authors. All authors contributed to the article and approved the submitted version.

## Conflict of Interest

The authors declare that the research was conducted in the absence of any commercial or financial relationships that could be construed as a potential conflict of interest.
